# Lectures on Surgical Pathology

**Published:** 1854-08

**Authors:** 


*Lectures on Surgical Pathology.* Delivered at the Royal College of Surgeons of England. By JAMES PAGET, F.R.S., &c. Philadelphia: Lindsay and Blakeston. 1854.

THE history of surgery illustrates the value of science in elevating art. The few simple operations performed in the earlier ages were attended with much danger, from the fact that anatomy as a science was imperfectly understood. In addition to this, the functions of parts, the mode of their growth, the condition of their life, and the laws of their repair, were almost unknown To the industry of the last and the first of the present century we are indebted for the most careful studies and the most minute descriptions in human anatomy. The first obstacle has been removed. To the present and the future we must look for a solution of those physiological questions so necessary to successful practice in this department of our profession.

Mr. Paget is a physiologist, and we are glad to see that he has made use of his store of knowledge in this department in these contributions to scientific surgery.

The first lecture of the course treats of nutrition, its nature, purpose, and conditions. The distinctions between growth and development are clearly and beautifully drawn. The renewal of structures by the removal of old and the formation of new particles, is illustrated by the manner in which the hair is reproduced. All the tissues are shown to have a definite length of life, after which they die, communicating that life, particle by particle, to a successor. In reference to this subject, our author says:

"I believe that we may assume an intimate analogy between the process of successive life and death, and life communicated to a successor, which is here shown, and that which is believed to maintain the ordinary nutrition of a part. It may be objected, indeed, that the death and casting out of the hair cannot be imitated in internal parts; but we are not without an example in which the absorption of a worn-out internal particle is exactly imitated in larger organs, at the end of their appointed period of life. I adduce the instance of the deciduous or milk-teeth.

"We trace each of these developed from its germ, and in the course of its development, seperating a portion of its capsule for the germ of its successor: then each, having gained its due perfection, retains for a time its perfect state, and still lives, though it does not grow. But at length, as the new tooth comes, the deciduous tooth dies, coincidently, not consequently; or rather, the crown of the old tooth dies, and is cast out like a dead hair; while its fang, with the bony sheathing, and the vascular and nervous pulp, degenerate, and are absorbed. It is here especially to be observed, that the degeneration is accompanied by some spontaneous transformation of the fang; for it could not be absorbed unless it were first so changed as to be soluble. And it is not degeneration, not death, which precedes its removal; for when a tooth-fang really dies, as that of the second tooth does in old age, then it is not absorbed, but is cast out entire as a dead part.

"Such, or nearly such, it is almost certain, is the process of nutrition everywhere: these may be taken as types of what occurs in other parts; for these are parts of complex organic structure and composition, and the teeth-pulps, which are absorbed as well as the fangs, are very vascular and sensitive; and, therefore, we may be nearly sure, are conformed to only the same laws as prevail in all equally organized parts.

"Nor are these the only instances that might be adduced. We see the like development, persistence for a time in the perfect state, death, and discharge, in all the varieties of cuticles, with which, also, we may connect the example of the gland cells; and in the epidermis we have, as in the teeth, an evidence of chemical change in the old cells, in the very different influence which acetic acid and potash exercise on them and on the younger cells, making these transparent, but leaving them scarcely changed.

"These things, then, seem to show that the ordinary course of each elementary organ in the body, after the attainment of its perfect state by development and growth, is to remain in that state for a time; then, independently of the death or decay of the whole body, and, at least in a great measure, independently of its own exercise or exposure to external violence, to die or to degenerate; and then, being cast out or absorbed, to make way for its successor.

"It appears, moreover, very probable that the length of life which each part is to enjoy is fixed and determinate, though, of course, in some degree subject to accidents which may shorten it, as sickness may prevent death through mere old age; and subject to the expenditure of life in the exercise of function. I do not mean that we can assign, as it is popularly supposed we can, the time that all our parts will last; nor is it likely that all parts are made to last an equal time, and then to be changed. The bones, for instance, when once completely formed must last longer than the muscles and other softer tissues. But, when we see that the life of certain parts is of determined length, whether they be used or not, we may assume, from analogy, the same of nearly all.

"For instance, the deciduous human teeth have an appointed duration of life—not, indeed, exactly the same in all persons, yet, on the whole, fixed and determinate. So have the deciduous teeth of other animals And, in all those numerous instances of periodical moulting, of shedding of the antlers, of the entire desquamation of serpents, and of the change of plumage in birds, and of the hair in mammalia. What means all this, but that these organs live their severally appointed times, degenerate, die, are cast away, and in due time are replaced by others; which, in their turn, are to be developed to perfection, to live their life in the mature state, and to be cast off? We may discern the same laws of life in some elementary structures; for example, in the blood-corpuscles, of which a first set, formed from embryo-cells, dsiappear at a certain period in the life of the embryo, being replaced and superseded by a second set, formed from lymph-corpuscles And in these also we may see an example of the length of life of elemental parts being determined, in some measure, by their activity in function; for if the development of the tad-pole be retarded by keeping it in a cold, dark place, and if, in this condition, the function of the first set of blood-corpuscles be slowly and imperfectly discharged, they will remain unchanged for even many weeks longer than usual. Their individual life will be thus prolonged, and the development of the corpuscles of the second set will be for the same time postponed.*

"The force of these facts is increased by the consideration of the exact analogy, the almost identity, of the processes of secretion and nutrition; for in no instance is the fact of this limited life of individual parts more clearly shown than in the gland-cells, by which periodical secretions are elaborated. The connecting link between such gland-cells and the most highly-organized parts, as well as a manifest instance of determinate length of life and natural death, is found in the history of the ova. These attain their maturity in fixed successive periods of days: they are separated (as the materials of secretions are) while yet living, and with a marvellous capacity of development, if only they be impregnated during the few days of life that remain to them after separation; but if these days pass, and impregnation is not effected, they die, and are cast out as impotent as the mere epithelial cell.

"Now, from these cases it is not by a far fetched analogy that we assume the like mortality in all other tissues, and that this is the principal source of impairment, and of change for the worse,

* See Kirke's Physiology, pp 65 and 290.

which every part of the body has within itself, even in the most perfect state, and in the conditions most favorable to life. And I may anticipate a future subject of consideration, by saying that the application of these truths is of some importance in practical pathology; inasmuch as the results of this degeneration of parts, at the close of their natural term of life, may be mingled with the effects of all the morbid processes by which the natural nutrition of a part is hindered or perverted. Hence, at least in part, the long continuing or permanent loss of power in an organ (say a muscle) which has been disused, or has been the seat of inflammation. This loss is not wholly due to a primary disease of the fibre: in part, it is because the inflammatory process and the organization of the morbid exudation exclude the ordinary process of nutrition; and the muscular fibres, which now, in the ordinary course of life, degenerate, are not replaced, or are imperfectly repaired."

The whole lecture is full of interest; but we have not space for further extract The conditions necessary to the normal nutrition of parts are discussed, namely—

1st. A right state of composition of the blood.

2d. A regular and not far distant supply.

3d. In most instances, at least, a certain influence of the nervous system.

4th. A natural state of the part to be maintained

By a right state of the blood, our author means that which is appropriate for the nourishment of the tissues at any given period in life, and not an absolute standard at each moment of our lives, the change going on in the organism gives rise to a change in the constitution of the blood. The growth of different structures, and the performance of the function of different organs, all modify it more or less.

From youth to age, therefore, through every hour of our being, this fluid differs, and the "right state" yesterday is not the right state to-day, nor will it be to-morrow. Each part, as it grows, abstracts a position from the circulating pabulum, returning to its elements, which have already subserved their purpose.

In Lecture Second, which treats of the conditions necessary to healthy nutrition, our author puts forth the bold proposition that each individual part, in its growth, abstracting from the blood those substances peculiar to itself, so far as every other tissue is concerned, a true excretion. The existence of rudimental organs as hair on the human fœtus, the mammary glands in the male &c., is accounted for on this principle. In their growth they serve the purpose of excreting organs, separating from the blood those substances which are rejected by every other structure. This idea will be better understood from the following extract:

"For these rudimental organs certainly do not serve, in a lower degree, the same purposes as are served by the homologous parts, which are completely developed in other species, or in the other sex. To say they are useless is contrary to all we know of the absolute perfection and all-pervading purpose of creation; to say they exist, merely for the sake of conformity, with a general type of structure, seems unphilosophical, while the law of the unity of organic types is, in larger instances, not observed, except when its observance contributes to the advantage of the individual. Rather, all these rudimental organs must, as they grow, be as excretions, serving a definite purpose in the economy by removing their appropriate materials from the blood, and leaving it fitter for the nutrition of other parts, or by adjusting the balance, which might else be disturbed by the formation of some other part. Thus they minister to the self-interest of the individual, while, as if for the sake of wonder, beauty, and perfect order, they are conformed with the great law of the unity of organic types, and concur with the universal plan observed in the construction of organic beings.

"And again—the principle that each organ, while it nourishes itself, serves the purpose of an excretion, has an application of peculiar interest in the history of development. For if it be the influential when all the organs are already formed, and are only growing or maintaining themselves, much more will it be so when the several organs are successively forming. At this time, as each nascent organ takes from the nutritive material its appropriate constituents, it will co-operate with the gradual self-development of the blood, to induce it in that condition which is essential, or more favorable, for the formation of the organs next in order to be developed."

Our author goes on to show that the existence in the blood of certain matters determines the formation of corresponding tissues. This is especially true of the lowly organized tissues.

These principles are applied to individual instances. "They suggest," says our author, that certain stand in their nutrition in a complemental relation to each other; so that neither of them can be duly formed or maintained in healthy structure, unless the right condition of the blood be induced and maintained by the formation of the other."

Not only so, but we may also believe, more particularly, "that certain organs are, mutually, as excretions from each other." This beautiful thought is in harmony with what we know of the economy of nature, and if true, as we believe it is, it possesses a practical value in the study of the pathology of the blood.

The subjects of formative process, growth, hypertrophy, atrophy, are discussed in a masterly style, and with the spirit of a true physiologist.

Lectures 7th, 8th, 9th, 10th, 11th an 12th treat of the repair and reproduction of injured and lost parts.

Inflammation and its attending phenomena and results occupy Lectures 13, 14, 15, 16, 17, 18 and 19. Lecture 21st is devoted to the consideration of specific diseases.

The larger portion of the work is devoted to the investigation of tumors, while the closing lecture treats of tubercle.

We have only room for a few extracts from that portion of the work devoted to the classification of tumors. Our author recognized the distinctions of malignant and non-malignant tumors. In addition to the general definition of tumors, which is as follows: parts which, 1st, are isolated from the surrounding parts by distinct investing layers of tissue; 2dly, though continuous with, the natural parts are abruptly circumscribed in the greater part of their extent; or, thirdly, are formed of new materials, infiltrated and growing in the interstices of natural parts. The following are given as characteristics of malignant growths:

"And, first, the intimate structure of malignant tumors is, usually, not like that of any of the fully developed natural parts of the body, nor like that which is formed in a natural process of repair or degeneration.

"Many of the cells of cancers, for example, may be somewhat like gland-cells, or like epithelium-cells; yet a practised eye can distinguish them, even singly. And much more plainly their grouping distinguishes them: they are heaped together disorderly, and seldom have any lobular or liminar arrangement, such as exists in the natural glands and epithelia, or in the innocent glandular or epithelial or epidermal tumors These innocent tumors are really imitations, so far as their structure is concerned, of the natural parts: and the existence of such imitations in any tumors make the diversity the heterology as it is called of the malignant tumors appear more evident

"Secondly. malignant growths may have the character of infiltration—*i e* their elementary structures may be inserted. infiltrated or diffused in the interspaces and cavities in which they lie. Thus in its early state a malignant tumor may comprise, with its own proper elements, those of the organ in which it is formed; and it is only in its later life that the elements of the tissue disappear from it. gradually degenerating, and being absorbed, or, possibly, yielding themselves as materials for its growth

"3d It is also generally characteristic of malignant tumors that they have a peculiar tendency to ulcerate, their ulceration being commonly preceded by softening One can indeed, in this particular, only observe a graduated difference between the innocent and the malignant diseases; for certain innocent tumors, if they grow very rapidly are apt very rapidly to decay; and they may suppurate and discharge their ichor and debris with foul and dangerous ulceration Thus the quickly-growing cartilaginous tumors may imitate in these respects malignant growths; so may large fibrous tumors when they soften and decay. Or, again, when an innocent tumor grows more rapidly than the parts over it can yield, they may waste and ulcerate, and allow it to protrude; and it may now itself ulcerate and look very malignant disease This may be seen in the protruding fibrous tumors that ulcerate and bleed; or, in a more striking manner, in the protruding vascular growths that have sprung up in the cystic tumors of the breast Or, once more, the characters of readiness to ulcerate may be imitated by innocent tumors after injuries, or in exposure to continued irritation; for they resist these things with less force than the similar natural parts do Hence, sloughing and ulcerating fibrous erectile, and other tumors have been often thought cancerous, and so described

"The respective tendencies to ulcerate can, therefore be counted only as constituting differences of degree between the innocent and the malignant tumors We may speak of a liability in the one case, of a proneness in the other

"4th The softening that often precedes the ulceration of malignant growths can hardly be considered separately from the minute account of their structure. I therefo e pass it by, and proceed to their fourth distinctive character, which is to be noticed in the modes of their ulceration.

"This is, that the ulcer which forms in, or succ[illegible] malignant growth, has no apparent disposition to heal; but a morbid substance, like that of which the original growth was composed, forms the walls or boundaries of the ulcer; and as this substance passes through the same process of ulceration which the primary growth passes through, so the malignant ulcer spreads and makes its way through tissues of all kinds.

"5th. Malignant tumors are, again, characterized by this: that they not only enlarge, but apparently multiply or propagate themselves; so that, after one has existed for some time, or has been extirpated, others like it grow, either in widening round its seat, or in parts more remote.

"8th. A sixth distinctive character of malignant tumors is, that, in their multiplication, as well as in their progress of ulceration, there is scarcely a tissue or an organ which they may not invade."

The physiology, as well as the anatomy of tumors claims a large share of the author's attention, and gives to the work its distinctive character.

We have seldom seen a book the perusal of which has given us so much pleasure, and we unhesitatingly recommend it to all who are interested in the application of physiology and surgical pathology.

For sale by D. B. Cooke & Co., 135 Lake st., Chicago.

